# A Prediction Model of the Concrete Cracking Induced by the Non-Uniform Corrosion of the Steel Reinforcement

**DOI:** 10.3390/ma13040830

**Published:** 2020-02-12

**Authors:** Wenjun ZHU, Kequan YU, Yude XU, Kai ZHANG, Xiaopei CAI

**Affiliations:** 1 Shanghai Key Laboratory of Rail Infrastructure Durability and System Safety, College of Transportation Engineering, Tongji University, Shanghai 201804, China; 2 Department of Civil and Environmental Engineering, University of Michigan, Ann Arbor, MI 48109, USA; kequany@umich.edu; 3 Guangdong Provincial Academy of Building Research Group Co., Ltd., Guangzhou 510500, China; miraclezheng@163.com; 4 Beijing Engineering and Technology Research Center of Rail Transit Line Safety and Disaster Prevention, School of Civil Engineering, Beijing Jiaotong University, Beijing 100044, China

**Keywords:** reinforced concrete, chloride-induced corrosion, non-uniform corrosion, analytical model, expansive stress

## Abstract

This paper investigates the influence of non-uniform corrosion in the transversal direction of the steel reinforcement on the cracking propagation of the concrete cover. An analytical model is proposed for the prediction of the corrosion-induced cracking performance. Both the thick cylinder theory of the concrete and the effect of transversal non-uniform corrosion of the steel reinforcement are involved by considering the corrosion layer of the corrosion products and a layer of concrete with the corrosion products filled with the pores. A three-stage corrosion-induced cracking of the concrete is proposed: corrosion without expansive stress to the concrete, corrosion with expansive stress to the adjacent concrete, as well as the corrosion-induced cracking of the concrete. By considering the non-uniform corrosion of the steel reinforcement and the tensile stress induced by the volumetric expansion of the corrosion products, the cracking initiation resulting from the non-uniform corrosion was involved in the prediction model. The models were also validated by the experimental results from both the corroded specimens and the existing literature, which would be helpful for the evaluation of the existing reinforced concrete constructions in the marine environment.

## 1. Introduction

Corrosion of steel reinforcement is usually considered as one of the most important factors for the deterioration of reinforced concrete (RC) elements [1], which threatens the serviceability and durability of the RC structures [2]. Especially for RC structures exposed to a marine environment, the wind could carry the chloride ions into the area as far as 3 km away from the sea [3,4]. The chloride ions penetrate the concrete gradually with an increase in the exposure period inevitably. Depassivation occurs to the steel reinforcement gradually and then followed by the corrosion of the steel reinforcement subsequently [5].

The corrosion products accumulate at the zone of the steel-concrete interface. However, the volume of the corrosion products is about 2–6 times larger than that of the original steel [6]. As a result, a volumetric expansion stress occurs to the concrete cover with the development of the corrosion. The corrosion-induced crack initiates gradually to the concrete cover when the tensile strength of the concrete is exceeded [7]. The corrosion-induced cracks provide a more convenient way for the accumulation of the aggressive agents around the steel reinforcement, which improves the corrosion process more seriously [8]. As a result, it will be very interesting to make clear the cracking process of the concrete cover due to the corrosion of the steel reinforcement in the RC structures.

Liu et al. [9] proposed a three-stage model of the corrosion-induced cracking process of the concrete cover: (1) the corrosion products penetrate into the pores at the steel-concrete interface during the initiation period and no additional stress happens to the concrete adjacent to the steel reinforcement; (2) the volumetric expansion results in the tensile stress to the concrete around the corrosion zone of the steel reinforcement when the pores adjacent to the corrosion zone are filled with the corrosion products; (3) the corrosion-induced cracking happens to the concrete cover when the tension strength of the concrete is overwhelmed, and the corrosion products fill into the corrosion-induced cracks gradually [10].

In the first stage, Michel et al. [11] investigated the penetration of the corrosion products into the concrete around the steel reinforcement based on the X-ray attenuation method and found a corrosion-accommodating region to hold the corrosion products during the time-dependent development. Wong et al. [12] also found a similar phenomenon and named it as the corrosion layer and corrosion product-filled pasted layer between the steel-concrete interface, respectively. The experimental results also found that the corrosion layer was smaller than 100 μm, and the corrosion product-filled pasted layer was usually smaller than 180 μm. Chitty et al. [13] verified the existence of the corrosion product-filled pasted layer in the concrete structures exposed to an aggressive natural environment [14]. In this paper, the corrosion layer and the corrosion product-filled pasted layer are adopted for the analytical model.

For the second stage and the third stage, the research mainly focuses on the development of the volumetric expansion stress between the steel-concrete interface and the propagation of the cracking process of the concrete cover [15]. The theory of mechanics is applied for the analytical method [16], by considering the corrosion products filling with the pores in the concrete around the corrosion zone of the steel reinforcement and accumulating in the steel-concrete interface simultaneously [17].

As for the third stage, the corrosion-induced cracks happen to the concrete cover and extended to the surface of the concrete cover gradually. Lu et al. [18] investigated the cracking process of the concrete cover with the corrosion product and proposed a prediction model based on the uniform corrosion according to the Faraday’s Law. In Lu et al.’s model, the corrosion-induced crack was supposed to be full of corrosion products. Similar results were also found in the available literature [12,19]. However, some experimental, such as the conclusions drawn by Michel et al. [20], also found that the corrosion-induced cracks were filled by the corrosion products only when the cracks had extended to the surface of the concrete cover. In this paper, both the empty cracks and the cracks filled with the corrosion products will be involved.

In this paper, the cracking path of the concrete cover induced by the non-uniform corrosion of the steel reinforcement will be studied. A typical model of non-uniform corrosion will be involved. The Timoshenko [21] theory will be adapted according to the hypothesis of the elastic behavior of the thick-walled cylinder. A new prediction model for the propagation of the cracking pattern of the concrete cover induced by the corrosion will be proposed.

## 2. Analytical Model

### 2.1. Non-Uniform Corrosion Model of the Steel Reinforcement

**Figure 1:**
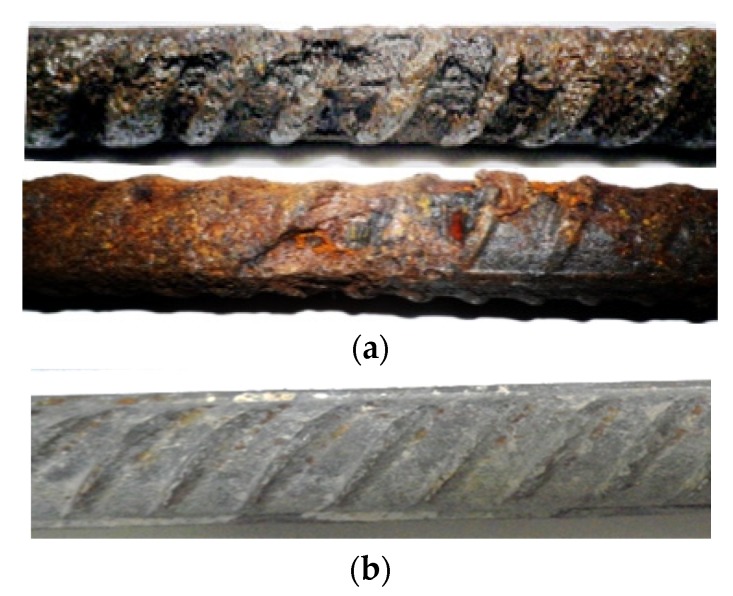
Corrosion of the steel reinforcement [22,23]. (**a**) Corrosion period of 26 years. (**b**) Non-corroded steel reinforcement.

According to the authors’ previous investigations on the corrosion of the steel reinforcement embedded in the RC beams exposed to a chloride environment for a long period of 26–28 years, the non-uniform corrosion occurred almost throughout the span of the length. More detailed information could be found in the published literature [22,23]. Figure 1 shows the image of the non-uniform corrosion by comparison with the non-corroded one [22]. The corrosion distributions in both the longitudinal and circumferential direction of the steel reinforcement are rather irregular, and no agreement has been reached up to now. However, it should be noted that only the non-uniform distribution of the transversal section of the steel reinforcement will be analyzed in this investigation. Up to now, several simplified models of the corrosion morphology in the cross-sectional image of the steel reinforcement are proposed and widely accepted, such as Melchers & Val’s model [24].

**Figure 2:**
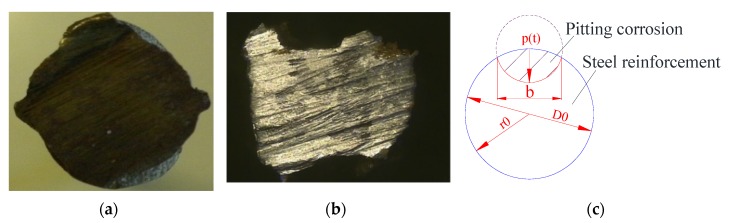
Simplification and comparison of the corrosion morphology [22,23]. (**a**) Non-corroded image. (**b**) Natural corroded image. (**c**) Melchers & Val’s model.

The typical corrosion morphology of Melchers & Val’s model was adopted in this investigation. Figure 2 shows the cross-sectional image of the typical corroded steel reinforcement with the corrosion age of 26 years, and the Melchers & Val’s model is also included to make the comparison. As shown in the figure, the pitting corrosion (non-uniform corrosion) occurs at the surface of the steel reinforcement. The cross-sectional loss of the pitting corrosion could be calculated as follows:

(1) $$A_{rs}=\{\begin{array}{cc} A_{1}+A_{2} & p\left(t\right)\leq \sqrt{2}r_{0}\\ \pi r_{0}^{2}-A_{1}+A_{2} & \sqrt{2}r_{0}<p\left(t\right)<2r_{0}\\ \pi r_{0}^{2} & p\left(t\right)\geq 2r_{0} \end{array}$$

where:

$$A_{1}=r_{0}^{2}\cdot arcsin\left(\frac{p\left(t\right)}{r_{0}}\sqrt{1-{\left(\frac{p\left(t\right)}{2r_{0}}\right)}^{2}}\right)-p\left(t\right)\cdot \sqrt{1-{\left(\frac{p\left(t\right)}{2r_{0}}\right)}^{2}}\cdot \left|r_{0}-\frac{p{\left(t\right)}^{2}}{2r_{0}}\right|$$

$$A_{2}=p{\left(t\right)}^{2}\cdot arcsin\left(\sqrt{1-{\left(\frac{p\left(t\right)}{2r_{0}}\right)}^{2}}\right)-\frac{p{\left(t\right)}^{3}}{2r_{0}}\cdot \sqrt{1-{\left(\frac{p\left(t\right)}{2r_{0}}\right)}^{2}}$$

$A_{rs}$ is the cross-sectional loss of the pitting corrosion;

$r_{0}$ is the radius of the origin steel reinforcement without corrosion;

p(t) is the maximum depth of the pitting corrosion.

**Figure 3:**
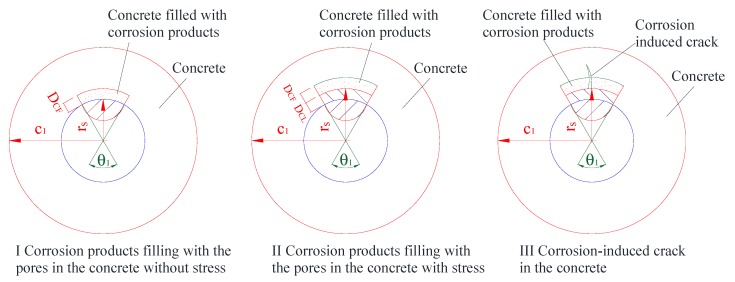
Three stages of the corrosion-induced cracks.

The corrosion morphology of Melchers & Val’s model will be adopted to analyze the mechanical performance of the corrosion-induced cracking of the concrete cover based on the knowledge of a thick-walled cylinder [20]. As investigated by Liu et al. [9] and Coccia et al. [25], three stages of the corrosion-induced cracking performance could be improved, as shown in Figure 3, where the pitting corrosion with the corrosion morphology of Melchers & Val’s model was involved.

According to Zhao et al. [26], the layer of the concrete filled with the corrosion products was developed with the corrosion propagation of the steel reinforcement in a linear way. The depth of concrete filled with the corrosion products was marked, as shown in Figure 3. The corrosion layer was marked as *D_CL_*, and the depth of the concrete with the pores filled with the corrosion products was marked as *D_CF_*. Zhao et al. [26] also proposed that the maximum values of *D_CL_* and *D_CF_* are usually considered to be 100 μm and 180 μm, respectively. The relationships of the values could be deduced as follows:

(2) $$\{\begin{array}{c} D_{CF}=n\cdot D_{CL},\;\;\;\;\;\;\;\;\;\;\;\;\;\;\;\;\;\;\;\;\;\;\;D_{CL}<D_{CL}^{cr}\\ D_{CF}=D_{CF}^{max}=n\cdot D_{CL}^{cr}\;\;\;\;\;\;\;\;\;\;D_{CL}\geq D_{CL}^{cr} \end{array}$$

where $D_{CF}^{max}$ is the maximum value of $D_{CF}$, $D_{CL}^{cr}$ is the threshold thickness of the corrosion layer corresponding to $D_{CF}^{max}$, and n is the ratio between *D_CF_* and *D_CL_*, as shown in Figure 3. According to Zhao et al. [26], n was supposed to be 1.8.

The area of the concrete filled with the corrosion products could be deduced by the pores of the concrete with the depth of *D_CF_*, which could be treated as the area of the corrosion products with the equivalent depth of $D_{CF}^{\prime }$ deduced as follows:

(3) $$D_{CF}^{\prime }=\frac{\emptyset \cdot \int_{0}^{\theta_{1}}D_{CF}r_{s}d\theta }{\theta_{1}r_{s}}=\;\emptyset \cdot D_{CF}$$

(4) $$\emptyset =\frac{\frac{w}{c}-0.36}{\frac{w}{c}+0.32}$$

where ∅ is the capillary porosity of the cementitious matrix around the steel reinforcement in the concrete;

$r_{s}$ is the internal radius of the cylinder, corresponding to the original radius of the steel reinforcement;

$\theta_{1}$ is the central angle corresponding to the corrosion zone around the steel reinforcement;

*w*/*c* is the ratio of the water to cement.

### 2.2. Corrosion Products Filled into the Pores of Concrete without Stress (Stage I)

**Figure 4:**
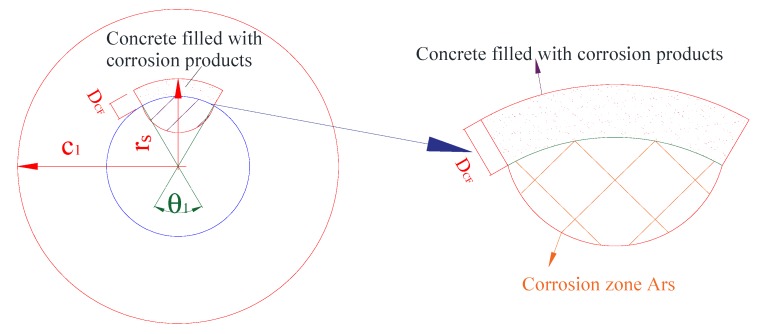
No stress thick-walled cylinder with non-uniform corrosion.

In the first stage, the corrosion occurs to the steel reinforcement gradually. Part of the corrosion products take the place of the corroded zone of the steel reinforcement. However, as the volume of the corrosion products is about 2–6 times of the steel reinforcement [6], the expansion volume of the corrosion products would migrate into the concrete adjacent to the steel-concrete interface within a certain distance of *D_CF_* as shown in Figure 4. As proposed by Zhao et al. [26], the depth of the concrete filled with the corrosion products was in a linear relationship with the corrosion degree of the steel reinforcement.

The area of the corrosion zone $A_{rs}$ would result in the area of corrosion products *A_cp_* as follow:

(5) $$A_{cp}=\alpha_{1}\cdot A_{rs}$$

where $\alpha_{1}$ is the volumetric expansion factor of the corrosion products, and in this paper, 3.75 is adopted based on Zhao et al. [26].

Then the area of corrosion products that migrate into the concrete from the steel-concrete interface during the stage I could be deduced as follows:

(6) $$D_{CF}^{\prime }=\;\emptyset \cdot D_{CF}=\left(\alpha_{1}-1\right)\cdot A_{rs}$$

### 2.3. Expansive Stress of the Concrete Cover Induced by the Corrosion Products (Stage II)

**Figure 5:**
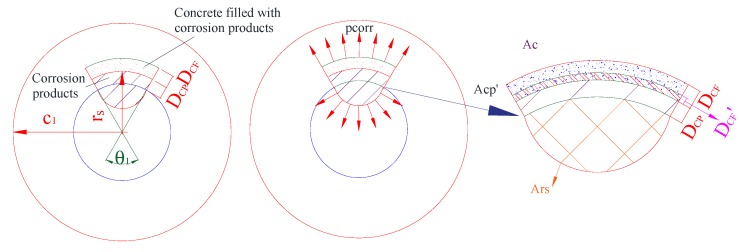
No cracked thick-walled cylinder with expansion stress.

In this stage, stress happened to the concrete adjacent to the steel-concrete interface, which was induced by the volumetric expansion of the corrosion products, as shown in Figure 5. Tension stress would be developed along the tangential direction of the corroded steel reinforcement in the cracking zone.

The stress could be treated as the contribution of the corrosion products layer with the depth of *D_CP_*, which could be deduced as follows:

(7) $$A_{cp}=\frac{1}{2}\theta_{1}\cdot {\left(r_{s}+D_{CP}\right)}^{2}-\frac{1}{2}\theta_{1}\cdot {\left(r_{s}\right)}^{2}$$

(8) $$A_{cp}=\left(\alpha_{1}-1\right)\cdot A_{rs}-\frac{1}{2}\theta_{1}\cdot {\left(r_{s}+D_{CP}+D_{CF}^{\prime }\right)}^{2}+\frac{1}{2}\theta_{1}\cdot {\left(r_{s}+D_{CP}\right)}^{2}$$

According to Equations (7) and (8), *D_CP_* could be expressed as follows:

(9) $$D_{CP}=-r_{s}-D_{CF}^{\prime }+\sqrt{{\left(r_{s}\right)}^{2}+\frac{\left(\alpha_{1}-1\right)\cdot A_{rs}}{2\theta_{1}}}$$

The hoop strain of the concrete corresponding to the corrosion zone could be expressed with the help of the radial displacement *D_CP_* as follows:

(10) $$\varepsilon_{rs}=\frac{\theta_{1}\cdot \left(r_{s}+D_{CP}\right)-\theta_{1}\cdot r_{s}}{\theta_{1}\cdot r_{s}}=\frac{D_{CP}}{r_{s}}$$

During the uncracked stage II, the corrosion pressure *p_corr_* corresponding to the corrosion zone could be evaluated according to Timoshenko [21]. In the hypothesis of elastic behavior of the cylinder and neglecting the Poisson effects, the pressure of a thick-walled cylinder subjected to an internal radial pressure could be expressed as follows:

(11) $$p_{corr\prime II}=E_{c}\varepsilon_{rs}\cdot \frac{\left(c_{1}^{2}-r_{s}^{2}\right)}{\left(c_{1}^{2}+r_{s}^{2}\right)}$$

(12) $$p_{t,o\prime II}=p_{corr}\cdot \frac{\left(c_{1}^{2}+r_{s}^{2}\right)}{\left(c_{1}^{2}-r_{s}^{2}\right)}$$

where

$p_{corr\prime II}$ is the corrosion pressure in the concrete at the radius *r_s_* along the radius direction at stage II;

$p_{t,o\prime II}$ is the hoop stress at the radius *r_s_* at stage II;

$E_{c}$ is the Young’s modulus of the concrete;

$p_{corr}$ is the corrosion pressure in the concrete at the radius *r_s_* along the radius direction;

*c_1_* is the external radius of the cylinder.

### 2.4. Cracking of the Concrete Cover Induced by the Corrosion Products (Stage III)

**Figure 6:**
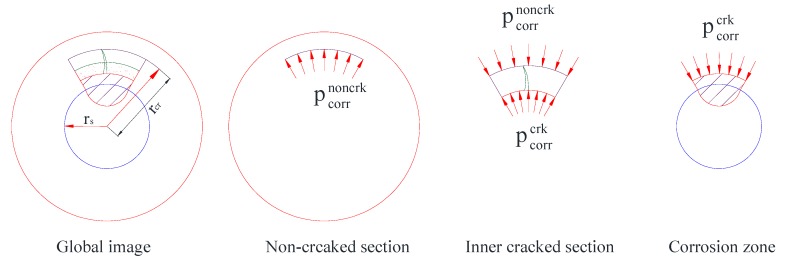
Cracked thick-walled cylinder with expansion stress.

The corrosion-induced crack happened to the concrete cover and developed gradually from the steel-concrete interface to the surface of the concrete. According to Tepfer [27], the cylinder could be subdivided into two sections, including the section with the steel reinforcement and the cracked concrete around the steel reinforcement. The area was determined by the cracking range *r_cr_*, as shown in Figure 6; the other section is the rest of the concrete in the non-cracked state which could still be treated to perform an elastic behavior.

The whole pressure stress induced by the corrosion products is mainly contributed by the cracked section $p_{corr}^{crk}$ and non-cracked section $p_{corr}^{noncrk}$ of the concrete cover, which can be expressed as follows:

(13) $$p_{corr\prime III}=p_{corr}^{noncrk}+p_{corr}^{crk}$$

As for the external section of the non-cracked cylinder, the elastic stress $p_{e}\;$ can be considered to be the same as the stage II. The internal stress of the non-cracked concrete cover can be deduced as follows:

(14) $$p_{e}=f_{ct}\left(\frac{c_{1}^{2}-r_{cr}^{2}}{c_{1}^{2}+r_{cr}^{2}}\right)$$

where $f_{ct}$ is the peak tensile stress of the concrete;

$\;r_{cr}$ is the critical radius corresponding to the internal radius of the non-cracked cylinder.

The contribution of the non-cracked section of the concrete could be achieved based on the equilibrium condition of the cracked cylinder as follows:

(15) $$p_{corr}^{noncrk}=\frac{r_{cr}}{r_{s}}\cdot p_{e}$$

The stress of the concrete in the corrosion-induced cracked section is exerted in a nonlinear way. The stress in the cracked concrete can be deduced from the radial pressure, which was deduced by Den et al. [28] as follow:

(16) $$p_{corr}^{crk}=\frac{1}{r}\int_{r}^{r_{cr}}\sigma_{t,crk\left(r\right)}\cdot dr$$

where $\sigma_{t,crk\left(r\right)}$ is the hoop stress at a generic radius *r*.

It should be noted that the soften branch is considered for the concrete’s constitutive tensile law. Den et al. [28] proposed that only the first branch of the softening curve is considered for the common concrete cover, which is also adopted in the following discussion of this investigation. The model proposed by Van [29] is considered as follow:

(17) $$\frac{\sigma_{t,crk\left(r\right)}}{f_{ct}}=a\cdot \frac{w}{w_{0}}+b$$

where *a*, *b* are the coordinates of the intersection point of the two softening lines. According to Bazant and the other available literature [30,31,32,33,34], $a_{1}$ = −2.27, $b_{1}$ = 1. *w* is the width of the corrosion-induced crack; $w_{0}$ is the peak of the corrosion-induced crack corresponding to a zone at a zero tensile strength, it is simply supposed to be 0.25 mm.

In order to make clear the relationship between the width of the corrosion-induced crack *w* and the radius *r*, the total elongation of the generic ring in the concrete cover was assumed to be constant, as proposed by Van [29]. The hoop strain of the concrete in the cracked section is the same as the strain corresponding to the peak tensile stress at the boundary of the concrete $\varepsilon_{cr}$. As a result, the total elongation $\Delta_{c}$ of the concrete cover at radius *r* can be deduced by:

(18) $$\Delta_{c}=n\cdot w\left(r\right)+\theta_{1}\cdot \varepsilon_{cr}\cdot r=\theta_{1}\cdot \varepsilon_{cr}\cdot r_{cr}$$

where *n* is the number of corrosion cracks.

The stress $p_{corr}^{crk}$ contributed by the concrete cover with corrosion-induced crack can be deduced from Equations (16)–(18):

(19) $$p_{corr}^{crk}=\frac{f_{ct}\cdot \varepsilon_{cr}\cdot \theta_{1}\cdot a_{1}\cdot r_{s}}{2n\cdot w_{0}}{\left(\frac{r_{cr}}{r_{s}}-1\right)}^{2}+f_{ct}\cdot b_{1}\cdot \left(\frac{r_{cr}}{r_{s}}-1\right)$$

The radial displacement of the concrete can be calculated by the integration of the hoop strain as follows:

(20) $$D_{cp\prime III}=\varepsilon_{cr}r_{cr}\left\{1+\left(\frac{c_{1}^{2}-r_{s}^{2}}{c_{1}^{2}+r_{s}^{2}}\right)ln\left(\frac{r_{cr}}{r_{s}}\right)+b_{1}\left[ln\left(\frac{r_{cr}}{r_{s}}\right)+\frac{r_{s}}{r_{cr}}-1\right]+\frac{\theta_{1}r_{cr}\cdot a_{1}\cdot \varepsilon_{cr}}{4n\cdot w_{0}}\left[2ln\left(\frac{r_{cr}}{r_{s}}\right)-{\left(\frac{r_{cr}}{r_{s}}\right)}^{2}+\frac{4r_{s}}{r_{cr}}-3\right]\right\}$$

The width of the corrosion-induced crack w(r) and the average value of the corrosion-induced crack $w_{m}$ could be deduced as follows:

(21) $$w\left(r\right)=\frac{\theta_{1}\cdot \varepsilon_{cr}}{n}\cdot \left(r_{cr}-r\right)$$

(22) $$w_{m}=\frac{w\left(r_{s}+D_{CP}\right)}{2}=\frac{\theta_{1}\cdot \varepsilon_{cr}\cdot \left(r_{cr}-r_{s}-D_{CP}\right)}{2n}$$

The corrosion-induced crack in the concrete cover was simplified to be in linear shape and the length $l_{cr}$ was calculated as follows:

(23) $$l_{cr}=r_{cr}-r_{s}-D_{CP}$$

As a result, the morphology of the corrosion-induced cracks of the concrete cover could be deduced according to the non-uniform corrosion of the steel reinforcement.

## 3. Model Validation

### 3.1. Corrosion of the Steel Reinforcement Corresponding to the Cracking of the Concrete Cover

The predicted results of the corrosion propagation of the steel reinforcement are conducted based on the theoretical method, as described in the previous section. The results of the previous experimental tests were retrieved from the available literature and then analyzed with the proposed theoretical model in order to make the validation of the theoretical model.

**Figure 7:**
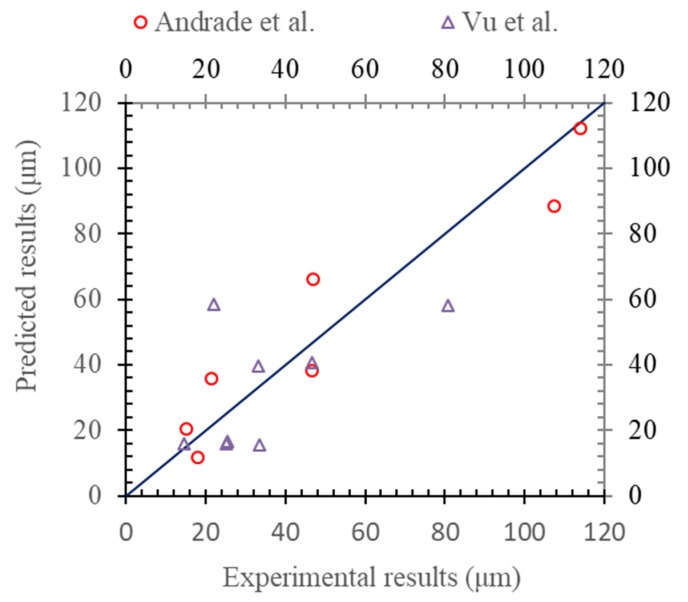
Radial loss corresponding to surface cracking (Melchers’ model).

The corrosion degree corresponding to the corrosion-induced cracking of the concrete cover was collected by Andrade et al. [35] and Vu et al. [36]. The experimental results about the depth of the radial loss due to the corrosion of the steel reinforcement were collected at the occurrence of the visible surface cracking on the concrete cover. The experimental results are compared with the predicted results, as shown in Figure 7.

**Figure 8:**
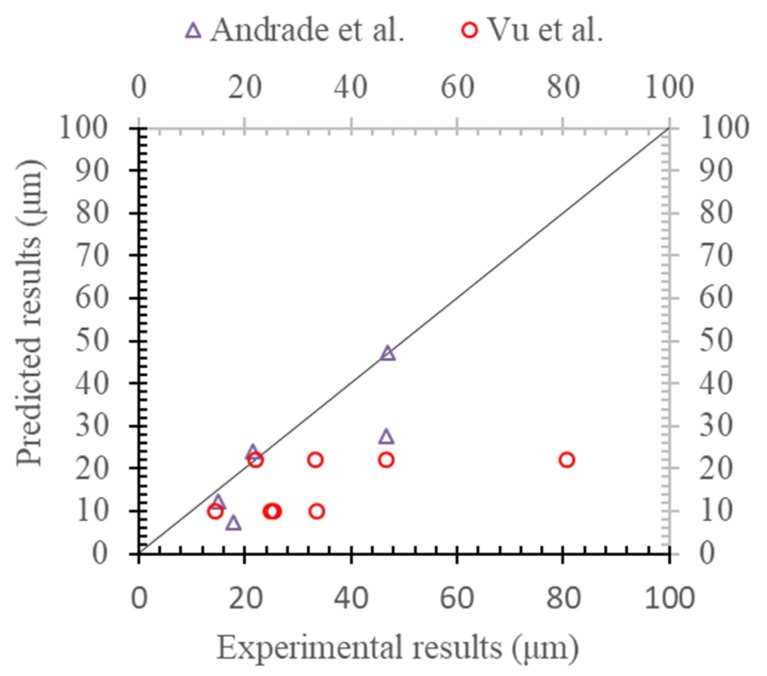
Radial loss with uniform corrosion model.

In fact, if the prediction results were deduced based on the uniform corrosion rather than the pitting corrosion of Melchers’ model, the experimental results were then compared, as shown in Figure 8. By comparing with that of Figure 7, it could be found that the prediction model based on the non-uniform corrosion of Melchers’ model was much better than that of the predicted results based on the uniform corrosion of the steel reinforcement.

### 3.2. Influence of Corrosion on the Propagation of the Cracking Path

**Figure 9:**
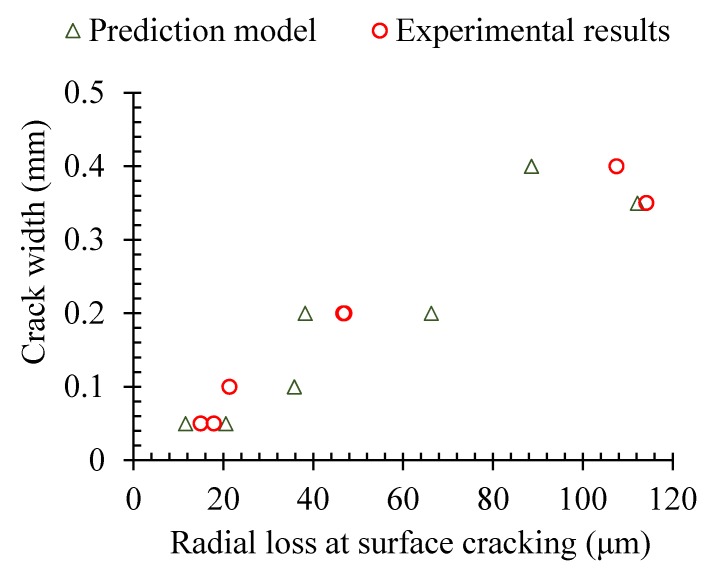
Relationship of the pitting corrosion and the crack width.

The relationships of the width of the first visible surface cracking in the concrete cover and the radial loss of the steel reinforcement due to the corrosion were also investigated by Andrade et al. [35]. The prediction results based on the proposed prediction model are compared with the experimental results, as shown in Figure 9.

It could be found that the results predicted by the proposed prediction model matched well with Andrade et al.’s experimental results [35]. When the width of the corrosion-induced cracks was smaller than 0.5 mm, the pitting depth of the steel reinforcement was in a linear relationship with the corrosion-induced cracks of the concrete cover.

## 4. Discussion

### 4.1. Compression Induced by the Corrosion of Steel Reinforcement

**Figure 10:**
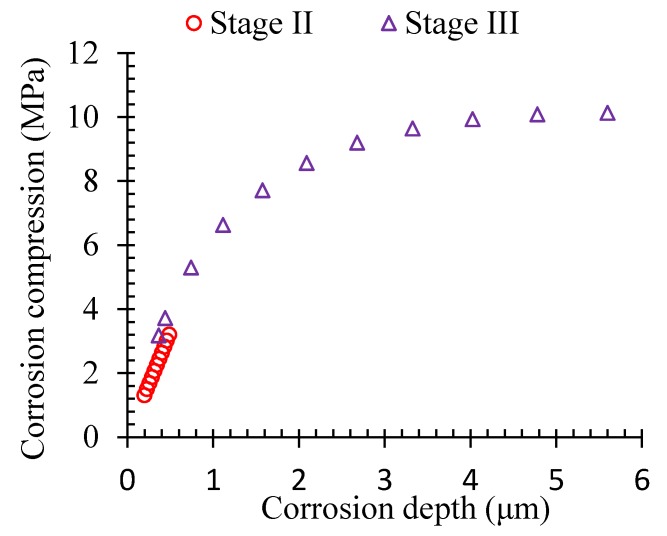
Displacement of the concrete in the steel-concrete interface.

The compression stress between the steel-concrete interface could be deduced based on the proposed theoretical model. Figure 10 shows the propagation of the maximum corrosion compression, which corresponded to the interface of the steel-concrete interface induced by the volumetric expansion of the corrosion products.

During Stage II, the corrosion compression was almost in linear relationship with the develop of the corrosion. However, the increase of the corrosion compression got reduced gradually during Stage III. The reason could be due to the fact that the inner cracks in the concrete cover could release the restraint for the deformation, and then the compression stress induced by the volumetric expansion of the corrosion products could be reduced even though the compression stress was still far smaller than the ultimate stress.

### 4.2. Radial Displacement of the Concrete in the Steel-Concrete Interface

**Figure 11:**
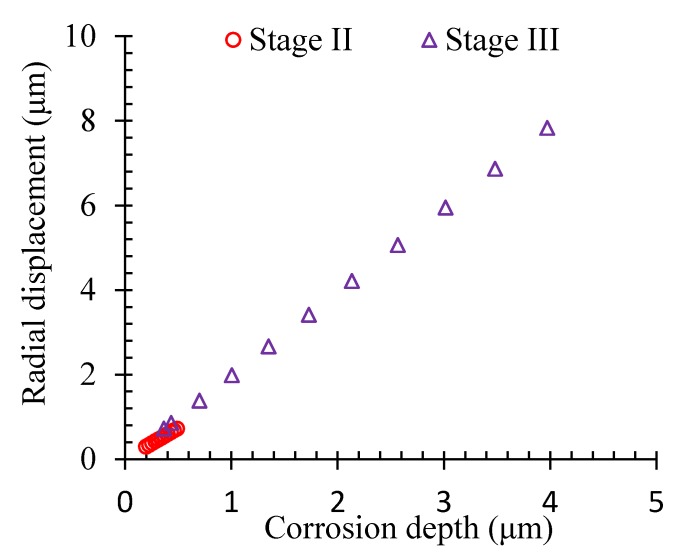
Displacement of the concrete at the steel-concrete interface.

The displacement happened to the concrete in the steel-concrete interface gradually once the volumetric expansion stress of the corrosion products was induced. Figure 11 shows the influence of corrosion on the radial displacement of the concrete in the interface. It could be found that the radial displacement got increased in a linear way with the corrosion depth. During Stage III, the increase of the radial displacement was slightly higher than that in Stage II due to the appearance of the corrosion-induced inner crack.

### 4.3. Propagation of the Inner Cracking

**Figure 12:**
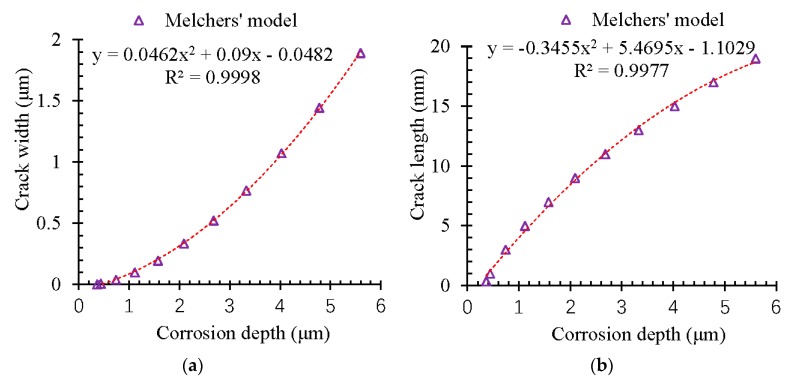
Propagation of the length of the corrosion-induced cracking path. (**a**)Propagation of the cracking width. (**b**) Propagation of the cracking length.

The propagation of the corrosion-induced crack in the concrete cover can also be investigated in this section based on the proposed prediction model. Before the corrosion-induced cracking happened to the surface of the concrete cover, the width and the length of the corrosion cracks in the inner concrete cover were assessed based on the pitting depth of the corrosion of the steel reinforcement. The results are shown in Figure 12.

According to Figure 12, both the width and length of the corrosion-induced cracks got developed with the propagation of the corrosion in a non-linear way. When the corrosion of the pitting depth reached 6 μm, the corrosion-induced cracks could be extended to be 20 mm, which corresponded to the minimum value of the concrete cover for most of the reinforced concrete structures and constructions. The width of the corrosion-induced crack was only 2 μm, which was still invisible.

**Figure 13:**
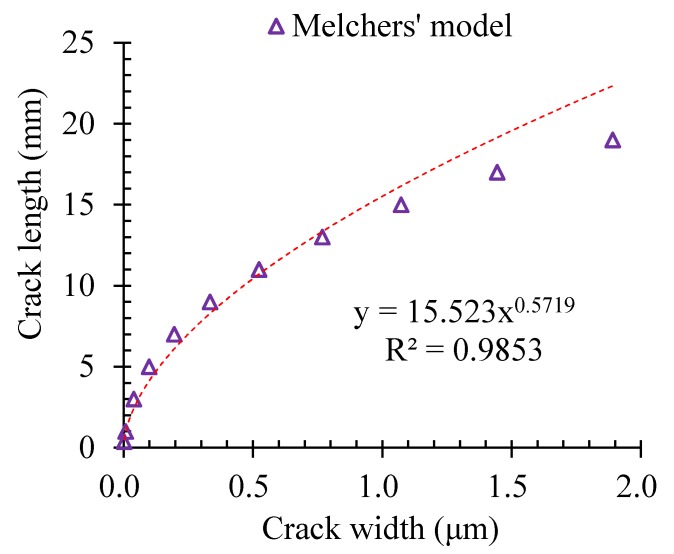
Relationship of the length and width of the corrosion-induced cracking path.

Moreover, the width of the corrosion-induced cracks developed in a much more significant way than that of the length. Figure 13 shows the relationships of the length and width of the corrosion-induced cracks. It could be found that the width of the corrosion-induced cracks got developed more significantly with the corrosion elapse.

## 5. Conclusions

The cracking performance of the concrete cover induced by the corrosion of the steel reinforcement was investigated in this paper. The thick cylinder theory was applied. The corrosion morphology of Melchers’ model was also involved in the theoretical analysis. The following conclusions could be drawn:

(1) A new predicted model about the cracking propagation of the concrete cover induced by the non-uniform corrosion of the steel reinforcement was proposed based on Melchers’ model and the thick cylinder theory. The predicted results were validated by the experimental results.
(2) The corrosion compression to the concrete was increased in a linear way at first. However, the appearance of the corrosion-induced cracks could release the compression significantly, even though the value was far smaller than the ultimate stress.
(3) With the propagation of the corrosion, the width and the length of the corrosion-induced crack were developed in a non-linear way in the concrete cover. The pitting depth of 6 μm would lead to an inner crack with a length of 20 mm and a width of about 2 μm in the concrete cover.

## Abbreviations

List of symbols

$A_{rs}$	cross-sectional loss of the pitting corrosion;
$r_{0}$	radius of the origin steel reinforcement without corrosion;
p(t)	maximum depth of the pitting corrosion;
*D_CL_*	depth of corrosion layer;
*D_CF_*	depth of concrete cover filled with the corrosion products;
$D_{CF}^{max}$	maximum value of *D_CF_*;
$D_{CL}^{cr}$	threshold thickness of corrosion layer;
$D_{CF}^{\prime }$	equivalent depth of the concrete cover;
∅	capillary porosity of cementitious matrix of the concrete cover;
$r_{s}$	internal radius of the cylinder;
$\theta_{1}$	central angle corresponding to the corrosion zone around the steel reinforcement;
*w*/*c*	ratio of water to cement;
*A_cp_*	area of corrosion products;
$\alpha_{1}$	volumetric expansion factor of the corrosion products;
$\varepsilon_{rs}$	tension strain of the concrete induced by the corrosion products;
*p_corr_*	corrosion pressure induced by the corrosion products;
$p_{corr}$	corrosion pressure in the concrete at the radius *r_s_* along the radius direction;
$p_{t}$	hoop stress at the radius *r_s_*;
*c_1_*	external radius of the cylinder;
$E_{c}$	Young’s modulus of concrete;
$p_{corr}^{crk}$	cracked section of the concrete cover;
$p_{corr}^{noncrk}$	non-cracked section of the concrete cover;
$p_{e}$	elastic stress of the concrete cover with inner crack;
$f_{ct}$	peak tensile stress of the concrete;
$r_{cr}$	critical radius corresponding to the internal radius of the non-cracked cylinder.
$\sigma_{t\prime crk\left(r\right)}$	hoop stress at a generic radius *r*;
*a*, *b*	coordinates of the intersection point of the two softening lines;
*w*	width of the corrosion-induced crack;
$w_{0}$	peak of the corrosion-induced crack corresponding to zero tensile strength;
$\Delta_{c}$	total elongation of the concrete cover;
*n*	number of corrosion cracks;
w(r)	width of the corrosion-induced crack;
$w_{m}$	average value of the corrosion-induced crack;
$D_{CP}$	deformation of the concrete induced by the corrosion products;
$\beta_{cr}$	ratio of the crack area filled with the corrosion products;
$\alpha_{p}$	ratio of hook stress and the radial stress;
